# Evaluating the Minimum Clinically Important Difference and Patient Acceptable Symptom State for the Womac Osteoarthritis Index after Unicompartmental Knee Arthroplasty

**DOI:** 10.3390/jcm12247618

**Published:** 2023-12-11

**Authors:** Umile Giuseppe Longo, Rocco Papalia, Stefano Campi, Sergio De Salvatore, Ilaria Piergentili, Benedetta Bandini, Alberto Lalli, Vincenzo Denaro

**Affiliations:** 1 Fondazione Policlinico Universitario Campus Bio-Medico, Via Alvaro del Portillo, 200, 00128 Roma, Italy; r.papalia@unicampus.it (R.P.); campi@gmail.com (S.C.); s.desalvatore@unicampus.it (S.D.S.); benedettabandini.000@gmail.com (B.B.); albertolalli30@gmail.com (A.L.); denaro.cbm@gmail.com (V.D.); 2 Research Unit of Orthopaedic and Trauma Surgery, Department of Medicine and Surgery, Università Campus Bio-Medico di Roma, Via Alvaro del Portillo, 21, 00128 Roma, Italy; 3 Research Unit of Ospedale Pediatrico Bambin Gesù, Dipartimento di Medicina e Chirurgia, Via della Torre di Palidoro, 00050 Fiumicino, Italy; 4 Consiglio Nazionale delle Ricerche—Istituto di Analisi dei Sistemi ed Informatica CNR-IASI, Laboratorio di Biomatematica, 00185 Roma, Italy; ilaria.piergentili94@gmail.com

**Keywords:** MCID, PASS, OKS, UKA, WOMAC osteoarthritis index, PROM

## Abstract

Patient-Reported Outcome Measures (PROMs) are standardized questionnaires that gather information on health-related quality of life directly from patients. Since a significant statistical mean change may not correspond to a clinical improvement, there is a need to calculate a considerable change in scores. This is done by the Minimum Clinically Important Difference (MCID) and Patient Acceptable Symptom State (PASS). The objective of this article is to report the MCID and the PASS values of the WOMAC (Western Ontario and McMaster University) osteoarthritis index for patients undergoing Unicompartmental Knee Arthroplasty (UKA). A total of 37 patients (25 females and 12 males; mean age 68 ± 8.1 years and mean BMI 28.7 ± 4) who underwent UKA were enrolled. All patients were assessed using the WOMAC and the Oxford Knee Score (OKS) questionnaires before and six months following the procedure. To measure the cut-off values for MCID, distribution methods and anchor methods were applied, while the PASS was assessed only via anchor approaches. The MCID related to the WOMAC average global score was 90.7 ± 7.6, the average pain dimension score was 93.2 ± 6.6, the average stiffness dimension score was 92.6 ± 17, and the average physical function dimension score was 89.7 ± 7.6. In terms of PASS, the normalized WOMAC was 82.8, the pain dimension was 87.5, the stiffness dimension was 93.7, and the functional dimension was 83.1. A 34.5 amelioration in the WOMAC score, from initial evaluation to final follow-up, using change in OKS > 5 as anchor, indicates that the patients’ health state improved to a clinically significant degree. A value at least of 82.8 in WOMAC score after treatment denotes that the symptom state is deemed acceptable by most of the patients.

## 1. Introduction

Unicompartmental Knee Arthroplasty (UKA) is the surgical choice for patients with isolated medial or lateral knee arthritis [1]. Over a decade in the United States, the revision rate of total knee arthroplasties increased by 5.4% [2]. However, it is correlated with a high revision rates [3].

Some patients report knee pain regardless the absence of radiographic signs of knee pathology [4]. For this reason, Patient-Reported Outcome Measures (PROMs) are regularly exploited in the literature to estimate the effectiveness of treatment [5].

PROMs are standardized questionnaires that gather information on surgical results including health-associated quality of life and performance status, directly from patients [6]. Contrarily to objective clinical outcome scales, which are assessed and reported by health-care providers, PROMs provide health status outcomes gathered from the patients’ own perspective, bypassing the clinician interpretation [7,8].

Three such are PROMs are WOMAC (Western Ontario and McMaster University) Osteoarthritis index, the Oxford Knee Score (OKS), and the Visual Analogue Scale (VAS) [9,10].

The WOMAC is a measure of symptoms and physical impairment originally designed for patients presenting with osteoarthritis (OA) of the hip or knee [11] by evaluating five pain-related activities, two stiffness categories, and 17 functional activities [11,12]. The OKS is a questionnaire for knee replacement patients that aims at demonstrating the patient’s assessment of the health status associated with their knee and the benefits of treatment [13]. The VAS, on the other hand, has been applied to quantify abstract and subjective measures such as standard of living, anxiety, and pain. The pain domain, for instance, is assessed by anchor descriptors such as “no pain” and “worst pain imaginable” [14].

Since a Mean Change (MC) can be statistically significant from preoperative to postoperative conditions, but may not correspond to a clinical improvement for the patient, there is a need to calculate a considerable change in scores. This could be determined by the Minimum Clinically Important Difference (MCID) [15]. The MCID could be defined as the smallest variation in PROMs score that reflects a clinically significant benefit increase from the patient’s perspective [16,17]. To assess the minimal PROM threshold corresponding to a satisfactory health for the patient, the Patient Acceptable Symptom State (PASS) is usually applied. The PASS is the threshold after which patients deem themselves satisfied [18]. To assess whether an acceptable symptom state is achieved at postoperative evaluations, anchor questions are exploited [19].

The PASS and MCID are different due to the fact that the first does not compare PROMs preoperatively to postoperatively, but rather determines a cross-sectional PROM value following the procedure [16].

These thresholds, in comparison to the objective clinical outcome scales, will make it easier to comprehend individual patient outcome data and provide doctors with the capacity to independently evaluate patient improvement and postoperative satisfaction levels. MCID and PASS serve as objective criteria, providing a standardized framework for evaluating the effectiveness of UKA therapies. This neutrality is critical for physicians in evaluating the surgery’s effectiveness across varied patient populations, resulting in more precise comparisons and evaluations of clinical outcomes, as well as a systematic approach in postoperative clinical monitoring [20,21].

In light of high revision rates and the complexities of PROM interpretation, the purpose of this study is to determine the importance of MCID and PASS in grasping individual patient outcome data. These measures enable operators to independently assess patient progress and postoperative satisfaction by offering a patient-centred perspective and a systematic approach to outcome assessment [21,22].

In the present literature, different studies have been carried out applying both the MCID and PASS to PROMs as the Oxford Knee Score (OKS), EQ-5D [23], and Forgotten Joint Score [19] following UKA, but no other research has identified such thresholds for the WOMAC.

Our hypothesis was that valid determinants of postoperative outcomes of the WOMAC score would be identifiable via MCID and PASS values.

## 2. Materials and Methods

### 2.1. Cohort Characteristics

Between January 2019 and October 2019, 37 patients (25 females and 12 males; mean age 68 ± 8.1 years and mean BMI 28.7 ± 4) who underwent UKA were retrospectively enrolled. All patients were evaluated using the WOMAC and the OKS questionnaires before the procedure and six months afterwards. All the procedures were performed by a single surgeon, consecutively, in a high-volume centre, within a public health setting.

All patients receiving UKA showed radiographic and clinical findings of unicondylar knee osteoarthritis (Kellgren–Lawrence Classification grades 3–4) [24]. Frontal varus–valgus <15°, flexion contracture <15°, functional integrity of anterior cruciate ligament, and absence of injuries of peripheral ligaments of the knee were the other inclusion criteria. Only UKAs with fixed bearing were performed. Only primary implants were considered. Revision surgeries and patients experiencing symptoms or signs of inflammatory arthritis were set as the exclusion criteria.

### 2.2. Assessment Instruments

The WOMAC is a disease-specific questionnaire that the patients can fill in autonomously. It is composed of 24 items, clustered within three dimensions: pain (5 questions), stiffness (2 questions), and physical function (17 questions). The Likert scale response was exploited to assess the following parameters through with five answer levels, corresponding to various levels of intensity: none (0), mild (1), moderate (2), severe (3), or extreme (4). The values at latest follow-up were calculated by summing the corresponding items for each parameter and standardizing to a range of values from 0 (worse condition) to 100 (best condition).

The Oxford Knee Score (OKS) is a value applied in daily orthopaedic clinical practice to gauge how well patients believe their knee-related health has improved since surgery. It is made of 12 questions, with answers ranging from 0 to 4 points on the Likert scale, which are added together in order to gather scores ranging from 0 to 48. Patients will be able to answer with the following options: non-existent (4), very mild (3), mild (2), moderate (1), or severe (0). High values of the score represent a good condition.

### 2.3. Statistical Analysis

With a Cohen’s effect size of 0.8, like in the literature [25,26], alpha = 0.05, two-tailed and power 80%, a cohort of 37 patients was considered essential.

Data normality for the WOMAC score and domains were determined by the Shapiro–Wilk test of normality. Given the non-normal distribution of the WOMAC and WOMAC domains, the Wilcoxon Signed Ranks Test was used to compare scores at baseline and at six months after the intervention. The correspondence between anchors was assessed with Spearman’s rho. The statistical significance threshold was set at 0.05. All statistical analyses were performed using IBM SPSS Statistics for Windows, version 26 (IBM Corp., Armonk, NY, USA), and the power analysis with G*Power 3.1.9.4.

### 2.4. Calculation of WOMAC Osteoarthritis Index MCID

To measure the cut-off values for MCID, various statistical tools were used to calculate both distribution methods and anchor methods.

The distribution-based approaches used were the 0.5 Standard Deviation (05 SD), related to a medium ES; the Standard Error of Measurement (SEM), corresponding to the amount of error related with a single subject assessment by the formula SEM = SD × √(1 − r), where SD is the standard deviation and r is the Cronbach’s alpha reliability coefficient in the samples included in the current study; and the Minimum Detectable Change (MDC), which constitutes the smallest variation above the measurement error with a 95% confidence interval assessment by the formula MDC= SEM × 1.96 × √2.

To use anchor approaches, all patients were required to assess their amelioration or decline by responding to a question about their amelioration or decline six months after UKA (How do you feel after the surgery carried out?). The five potential answers were “much better”, “somewhat better”, “equal”, “somewhat worse”, and “much worse”. Patients who responded “equal”, “somewhat worse”, and “much worse” were judged “No responders”, while patients who responded “somewhat better” were judged “Minimally improved”.

An additional anchor was exploited to evaluate the consistency of the outcomes; specifically, a change in OKS at least 5 between pre-operative to six months follow-up. Therefore, patients with a change in OKS at least of 5 were defined “Minimal improved”. The change in OKS of at least 5 was chosen because 5 was the MCID of OKS [27]. An external anchor was judged as valid if the correlation coefficients with WOMAC and WOMAC domains were at least 0.3 with *p* < 0.05.

Two anchor approaches were used. First, the MC score for individuals who stated that changes were “somewhat better” in the improvement question, calculated by dividing the score at baseline by the difference at six months. Second, the Receiver Operating Characteristics (ROC) curve approach, comparing the two groups “No responders” and “Minimal improved”, was used. The Youden index was applied as optimal cut-off value of each dimension.

### 2.5. Calculation of WOMAC Osteoarthritis Index PASS

An anchor for PASS that takes pain, physical function, and patient satisfaction into account is valid [28]. The following query was proposed by Kvien et al. as anchor for PASS “Taking into account all the activities you have during your daily life, your level of pain, and also your functional impairment, do you consider that your current state is satisfactory?” [28].

To calculate WOMAC’s PASS the following question was applied: “In general would you say that your health is at least good?”, with potential responses “Yes” or “No”. To evaluate the consistency of outcomes, a different anchor was used: high value of OKS at latest follow-up. Since OKS score between 40 to 48 represents “satisfactory joint function” [29], a cut-off of 40 was chosen. Therefore, patients with OKS at least 40 were considered “responders”. The ROC curve point where the cut-off was determined using the Youden index and the cumulative percentage curve’s 75th percentile of patients who consider they are in an acceptable state of symptoms were used to determine the WOMAC PASS thresholds.

The authors considered statistically significant to be *p* < 0.05.

## 3. Results

A total of 37 patients who underwent UKA were enrolled. The Shapiro–Wilk test showed no-normality of WOMAC distribution at baseline and six months postoperative. Since the correlation between WOMAC (and WOMAC domains) and OKS was higher than 0.3, OKS was defined as valid external anchor for MCID and PASS (Table 1 and Table 2). Since the Area Under the Curve (AUC) using the question about improvement was always less than 0.7, this anchor was not valid.

### 3.1. MCID of WOMAC Osteoarthritis Index

#### 3.1.1. Global WOMAC Osteoarthritis Index Score

The average WOMAC score improved from 58.4 ± 10.2 at baseline to 90.7 ± 7.6 at six months postoperative follow-up. A statistically significant variation between initial evaluation and six months follow-up was found (*p* < 0.001).

Table 3 exhibits data on the MCID in the WOMAC score. A score of 14.8 corresponds to the smallest variation in WOMAC score which accounts for actual change rather than measurement inaccuracy. Using change in OKS > 5 as anchor, the MCID calculated with MC was 34.5, while the MCID calculated with ROC method was 4.7 (AUC = 0.9).

#### 3.1.2. Pain Dimension of WOMAC Osteoarthritis Index

The average pain dimension of WOMAC score improved from 56.4 ± 13 at baseline to 93.2 ± 6.6 at six months postoperative follow-up. A statistically significant variation between preoperative and six months follow-up was found (*p* < 0.001).

Table 4 shows data on the MCID in the Pain dimension. The 0.5 SD was 7.7, the SEM was 9.8, and the MDC was 27.2. Thus, 27.2 corresponds to the minimum change in WOMAC score that reflects true change rather than a measurement error.

#### 3.1.3. Stiffness Dimension of WOMAC Osteoarthritis Index

The average stiffness dimension of WOMAC score improved from 60.1 ± 27 at initial evaluation to 92.6 ± 17 at six months postoperatively. A statistically significant variation between preoperative and six months follow-up was found (*p* < 0.001).

Table 5 exhibits values on the MCID in the Stiffness dimension. A score of 29.5 corresponds to the lowest change in WOMAC score which accounts for actual change rather than measurement inaccuracy.

#### 3.1.4. Physical Function Dimension of WOMAC Osteoarthritis Index

The average Physical Function dimension of WOMAC score improved from 58.8 ± 10 at baseline to 89.7 ± 7.6 at six months postoperative evaluation. A statistically significant variation between preoperative and six months follow-up was found (*p* < 0.001).

Table 6 exhibits data on the MCID in the Physical Function parameter. A score of 16.6 corresponds to the lowest change in WOMAC score which accounts for actual change rather than measurement inaccuracy.

### 3.2. PASS of WOMAC Osteoarthritis Index

PASS calculated for normalized WOMAC in patients who received UKA ranged from 82.8 to 95.1. The cut-off values computed with 75th percentile approach were 95.1 and 91.7. PASS calculated for pain in WOMAC ranged from 87.5 to 95. PASS calculated for stiffness in WOMAC ranged from 93.7 to 100. Finally, PASS calculated for physical function in WOMAC ranged from 83.1 to 91.2 (Table 7).

## 4. Discussion

The main finding of the present study is that, using only the valid anchor, the WOMAC’s MCID in patients undergoing UKA ranged between 4.7 to 34.5 points. The lowest value was derived from the ROC calculation (with AUC = 0.9), whereas the greatest value resulted from the MC calculation with variation in OKS > 5 as anchor.

An MCID of 7.1 was obtained using a medium ES (ES of 0.5) assumption (0.5 SD). The SEM came in at 5.3. With a 95% degree of confidence, the lowest variation considerable as above the measurement error was 14.8 (MDC).

Therefore, it can be said that, with a probability of 95%, a change in score higher than 14.8 is not given by measurement errors but by a real improvement in the patient.

Anchor approaches and distribution were used to calculate MCID of WOMAC. The change in OKS > 5 and the question “How do you feel after the procedure you have undergone?” were used as anchors. The question about improvement of patients was not a valid anchor because the AUC was always less than 0.7.

To the authors’ understanding, no previous studies assessing the MCID for WOMAC are present in the current literature. Rather, only MCIDs of OKS and SF-36 were reported [30,31,32].

However, different articles studied the WOMAC MCID in patients that received Total Knee Arthroplasty (TKA): Quintana et al. described an MCID for the WOMAC of 22.60 for pain, 17.67 for functional limitations, and 12.94 for stiffness [33]. Also, in the study of Escobar et al., WOMAC’s MCID of 22.39 for pain, 13.11 for functional limitations, and 29.12 for stiffness [34] were calculated. Within the same context, Maratt et al. found a MCID for WOMAC of 31.25 for pain, 25.00 for stiffness, and 26.93 for functional limitations [35]. Clement et al. reported values for the MCID for the total WOMAC score ranging from 17 to 20 [17].

According to Copay et al. definition, the two requirements for the validity of MCID were that it needs to be correlated to the patient impression of significance of change, and must be not less nor equal than the measurement error [36]. Therefore, based on these criteria, MC seems to be the most suitable tool to define the MCID in the current study. The MCID assessed with MC method resulted to be higher than SEM. Methods 0.5 SD and MDC were also higher than SEM, but these anchoring approaches are usually applied in more recent orthopaedic publications [37,38,39].

The same considerations were made for the WOMAC’s dimension. Comparing with the literature, the most appropriate WOMAC’s MCID for the pain dimension is 40 [35], for the stiffness dimension is 31.2 [34,35] and for the functional dimension is 28.7 [35]. The most suitable MCID of total WOMAC is 34.5 [40,41].

Additionally, a further significant finding of the current study involved the WOMAC’s PASS: in the present study, the PASS values ranged between 82.8 and 95.1. Given the current literature available on the topic, the most valid PASS value for WOMAC in the current study appears to be 82.8, the one calculated with the ROC method and the question about health as anchor.

To the authors’ knowledge, only one article calculating the WOMAC’s PASS in patients undergoing UKA has been published. Wang et al. [42], in the aforementioned study, reported a WOMAC’s PASS value of 81.77. However, this article and the current study differ in terms of mean follow-up, resulting in 21.5 months and six months postoperatively, respectively.

Furthermore, in the current study, the WOMAC’s PASS value for pain and stiffness resulted in 87.5 and 93.7, respectively. In terms of functional limitations, the assessed value was 83.1.

Floor and ceiling effects were very low (0%) in the global WOMAC score. On the pain and stiffness dimension, a notable ceiling effect at last follow-up was found (24.3% and 78.4%, respectively). These results are in line with those found in Gandek et al. [43].

Finally, understanding clinically meaningful improvement in patients is intricate, influenced by factors like expectations and mood. Hence, the capability to translate changes in PROMs or absolute values into clinically meaningful success is crucial for assessing intervention effectiveness and guiding patients with realistic expectations [44].

The present article relies on several points of strengths. This is the only publication reporting both the MCID and PASS for WOMAC in patients that have undergone a UKA. Additionally, the score exploited as anchor is valid and regularly applied in similar studies. Furthermore, the commonly accepted ad hoc methods were exploited to determine MCID and PASS. Furthermore, since two anchors were exploited for both MCID and PASS in the present study, uniformity of outcomes across multiple anchors was reported. Finally, the results considered both the global WOMAC its dimensions: pain, stiffness, and functional limitations.

However, this paper also has limitations. First, the mean pre- and postoperative follow-up was six months, therefore making it impossible to deliver objective information on long-term WOMAC scores. There may be a correlation between MCID values and follow-up time, which may affect the results in a way that is not considered in this study. Secondly, even if, according to the power analysis, the cohort was sufficient in terms of quantity, a higher number of patients has been commonly taken into consideration in similar literature.

Moreover, the inconsistency in representing measurement values within our study stems from variations in how values are presented across source articles. This variability poses a challenge to seamless data integration. Additionally, the absence of graphical representations, beyond the proposed tables, should be noted as a limitation in providing a more comprehensive statistical overview.

## 5. Conclusions

A 34.5 improvement on WOMAC score, from baseline to six months postoperatively, indicates that patients have managed to achieve a clinically relevant amelioration in terms of health status. A value at least of 82.8 in WOMAC score after treatment indicates that symptom state can be deemed acceptable by most patients. These cut-offs will aid in the comprehension of individual patient outcome data by providing doctors with the freedom to independently evaluate patient improvement and satisfaction levels post-UKA.

## Figures and Tables

**Table 1:** Correlation between WOMAC osteoarthritis index change, change in domains of WOMAC osteoarthritis index, and change in OKS for MCID.

Score	Correlation with OKS	*p*-Value
global WOMAC	0.7	<0.001
pain	0.5	0.003
stiffness	0.5	0.002
functional	0.6	<0.001

WOMAC, Western Ontario and McMaster University osteoarthritis index; OKS, Oxford Knee Score.

**Table 2:** Correlation between WOMAC osteoarthritis index at last follow-up, WOMAC osteoarthritis index domains at last follow-up, and OKS at last follow-up for PASS.

Score	Correlation with OKS	*p*-Value
global WOMAC	0.6	<0.001
pain	0.5	0.004
stiffness	0.5	0.001
functional	0.6	<0.001

WOMAC, Western Ontario and McMaster University osteoarthritis index; OKS, Oxford Knee Score.

**Table 3:** MCID of WOMAC osteoarthritis index score.

MCID	Cut-Off Value	Anchor
0.5 SD	7.1	/
SEM	5.3	/
MDC	14.8	/
ROC (AUC)	42.2 (0.5)	Question about improvement
ROC (AUC)	4.7 (0.9)	Change OKS > 5
MC	28.3	Question about improvement
MC	34.5	Change OKS > 5

MCID, Minimum Clinically Important Difference; SD, Standard Deviation; SEM, Standard Error of Measurement; MDC, Minimum Detectable Change; ROC, Receiver Operating Characteristics; AUC, Area Under the Curve; MC, Mean Change; OKS, Oxford Knee Score.

**Table 4:** MCID of WOMAC Pain dimension.

MCID	Cut-Off Value	Anchor
0.5 SD	7.7	/
SEM	9.8	/
MDC	27.1	/
ROC (AUC)	42.5 (0.6)	Question about improvement
ROC (AUC)	15 (0.9)	Change OKS > 5
MC	35.3	Question about improvement
MC	40	Change OKS > 5

MCID, Minimum Clinically Important Difference; SD, Standard Deviation; SEM, Standard Error of Measurement; MDC, Minimum Detectable Change; ROC, Receiver Operating Characteristics; AUC, Area Under the Curve; MC, Mean Change; OKS, Oxford Knee Score.

**Table 5:** MCID of WOMAC Stiffness dimension.

MCID	Cut-Off Value	Anchor
0.5 SD	17.5	/
SEM	10.7	/
MDC	29.5	/
ROC (AUC)	62.5 (0.3)	Question about improvement
ROC (AUC)	31.2 (0.7)	Change OKS > 5
MC	26.3	Question about improvement
MC	35.3	Change OKS > 5

MCID, Minimum Clinically Important Difference; SD, Standard Deviation; SEM, Standard Error of Measurement; MDC, Minimum Detectable Change; ROC, Receiver Operating Characteristics; AUC, Area Under the Curve; MC, Mean Change; OKS, Oxford Knee Score.

**Table 6:** MCID of Physical Function dimension.

MCID	Cut-Off Value	Anchor
0.5 SD	6.9	/
SEM	6	/
MDC	16.6	/
ROC (AUC)	43.4 (0.4)	Question about improvement
ROC (AUC)	28.7 (0.9)	Change OKS > 5
MC	26.5	Question about improvement
MC	32.8	Change OKS > 5

MCID, Minimum Clinically Important Difference; SD, Standard Deviation; SEM, Standard Error of Measurement; MDC, Minimum Detectable Change; ROC, Receiver Operating Characteristics; AUC, Area Under the Curve; MC, Mean Change; OKS, Oxford Knee Score.

**Table 7:** PASS of WOMAC osteoarthritis index.

Score	Anchor	ROC	(AUC)	75th Percentile
global WOMAC	Question about health	82.8	0.9	95.1
pain		87.5	0.9	90
stiffness		93.7	0.9	100
functional		83.1	0.9	90.4
global WOMAC	OKS > 40	91.1	0.8	91.7
pain		92.5	0.8	95.0
stiffness		93.8	0.8	100
functional		89	0.8	91.2

WOMAC, Western Ontario and McMaster University osteoarthritis index; OKS, Oxford Knee Score; ROC, Receiver Operating Characteristics; AUC, Area Under the Curve.

## Data Availability

The data presented in this study are available on request from the corresponding author.
